# Preliminary evidence of content validity with elderly people of a social cognition assessment instrument

**DOI:** 10.1002/alz70861_108695

**Published:** 2025-12-23

**Authors:** Luiz Alves Ferreira Júnior, Beatriz Figueiredo e Silva, Jéssica Diniz Ferreira, Amanda Pelinson Gomes Tiago, Júlia Costa C Dias, Marcela Sena Braga, Debora Marques de Miranda, Marco Aurelio Romano‐Silva, Maria Aparecida Camargos Bicalho, Jonas Jardim de Paula, Bernardo de Mattos Viana

**Affiliations:** ^1^ Older Adult’s Psychiatry and Psychology Extension Program (PROEPSI), School of Medicine, Universidade Federal de Minas Gerais (UFMG), Belo Horizonte, Minas Gerais Brazil; ^2^ Universidade Federal de Minas Gerais, Belo Horizonte, Minas Gerais Brazil; ^3^ Universidade Federal de Minas Gerais, Belo Horizonte Brazil; ^4^ Faculty of Medical Sciences of Minas Gerais, Belo Horizonte, Minas Gerais Brazil; ^5^ Molecular Medicine Postgraduate Program, School of Medicine, Universidade Federal de Minas Gerais (UFMG), Belo Horizonte, Minas Gerais Brazil; ^6^ Cog‐Aging Research Group, Universidade Federal de Minas Gerais (UFMG), Belo Horizonte, Minas Gerais Brazil; ^7^ Older Adult’s Psychiatry and Psychology Extension Program (PROEPSI), School of Medicine, Universidade Federal de Minas Gerais (UFMG), Belo Horizonte, 3013010 Brazil; ^8^ Federal University of Minas Gerais, Belo Horizonte Brazil; ^9^ Universidade Federal de Minas Gerais, Belo Horizonte, 3013010 Brazil; ^10^ Neurotec R National Institute of Science and Technology (INCT‐Neurotec R), Faculty of Medicine, Universidade Federal de Minas Gerais (UFMG), Belo Horizonte, Minas Gerais Brazil; ^11^ Molecular Medicine Program, School of Medicine, Federal University of Minas Gerais, Belo Horizonte, Minas Gerais Brazil; ^12^ Department of Psychiatry, School of Medicine, Federal University of Minas Gerais, Belo Horizonte, Minas Gerais Brazil; ^13^ Cog‐Aging Research Group, Belo Horizonte, Minas Gerais Brazil; ^14^ Graduate Program in Applied Sciences to Adult Health, Faculdade de Medicina, Universidade Federal de Minas Gerais, Belo Horizonte Brazil; ^15^ UFMG, Belo Horizonte Brazil; ^16^ INCT – NeuroTecR and CTMM, Belo Horizonte, Minas Gerais Brazil; ^17^ Geriatrics and Gerontology Center Clinical Hospital of University of Minas Gerais, Belo Horizonte, Minas Gerais Brazil; ^18^ Sciences Applied to Adult Health Postgraduate Program, School of Medicine, Universidade Federal de Minas Gerais (UFMG), Belo Horizonte, Minas Gerais Brazil; ^19^ Federal University of Minas Gerais, Belo Horizonte, Minas Gerais Brazil; ^20^ Older Adult’s Psychiatry and Psychology Extension Program I Federal University of Minas Gerais, Belo Horizonte, MG Brazil; ^21^ National Institute of Science and Technology (INCT‐Neurotec R), Faculty of Medicine, Federal University of Minas Gerais, Belo Horizonte, Minas Gerais Brazil; ^22^ Older Adult’s Psychiatry and Psychology Extension Program Federal University of Minas Gerais, Belo Horizonte, Minas Gerais Brazil

## Abstract

**Background:**

Social cognition refers to mental processes involved in interpreting social and emotional information, which help individuals respond to others’ intentions and behaviors. In Brazil, there are few instruments to assess this construct, especially for older adults. Objective: To investigate evidence of content validity with the target population of an instrument designed to assess social cognition in older adults and to present possible revisions to the instrument under development.

**Method:**

The instrument is based on Theory of Mind (ToM) tasks and includes three parts: False Belief, Strange Stories, and Faux‐Pas. Each part has four adaptive items (12 in total), each composed of a vignette describing a situation in which the participant must identify a social cognition issue. Each item includes 6 to 8 questions, totaling 80. A pilot sample of 14 older adults (mean age = 68 ± 6.4 years; 11 women; mean education = 9.9 ± 4.9 years) completed the tasks. Content validity was analyzed qualitatively, based on accuracy rates for each question and item.

**Result:**

The mean accuracy across questions was 79.9% ± 23, with a range from 7.1% to 100%. At the item level, mean accuracy was 82.3%. Only one item scored 50%; all others had at least 77.7%. Faux‐pas items were the most challenging. The item with the lowest accuracy appeared to have issues with its vignette, as many participants did not perceive the situation as a social faux‐pas. Among the questions, the most difficult involved inferring the speaker’s motivation related to the faux‐pas.

**Conclusion:**

The instrument was generally well understood by the target population, based on accuracy rates. Some modifications may enhance item clarity for older adults. Future steps include investigating criterion validity in clinical samples and developing normative data.

